# The VIM-AS1/miR-655/ZEB1 axis modulates bladder cancer cell metastasis by regulating epithelial–mesenchymal transition

**DOI:** 10.1186/s12935-021-01841-y

**Published:** 2021-04-26

**Authors:** Yaoyao Xiong, Xiongbing Zu, Long Wang, Yuan Li, Minfeng Chen, Wei He, Lin Qi

**Affiliations:** 1 Department of Urology, Xiangya Hospital, Central South University, No. 87 Xiangya Road, Kaifu District, Changsha, 410008 Hunan China; 2 Department of Cardiopulmonary Bypass, The Second Xiangya Hospital, Central South University, Changsha, 410011 Hunan China

**Keywords:** Bladder cancer, VIM-AS1, Epithelial–mesenchymal transition (EMT), miR-655, ZEB1

## Abstract

**Background:**

Invasive bladder tumors cause a worse prognosis in patients and remain a clinical challenge. Epithelial–mesenchymal transition (EMT) is associated with bladder cancer metastasis. In the present research, we attempted to demonstrate a novel mechanism by which a long noncoding RNA (lncRNA)-miRNA-mRNA axis regulates EMT and metastasis in bladder cancer.

**Methods:**

Immunofluorescence (IF) staining was used to detect Vimentin expression. The protein expression of ZEB1, Vimentin, E-cadherin, and Snail was investigated by using immunoblotting assays. Transwell assays were performed to detect the invasive capacity of bladder cancer cells. A wound healing assay was used to measure the migratory capacity of bladder cancer cells.

**Results:**

Herein, we identified lncRNA VIM-AS1 as a highly- expressed lncRNA in bladder cancer, especially in metastatic bladder cancer tissues and high-metastatic bladder cancer cell lines. By acting as a ceRNA for miR-655, VIM-AS1 competed with ZEB1 for miR-655 binding, therefore eliminating the miR-655-mediated suppression of ZEB1, finally promoting EMT in both high- and low-metastatic bladder cancer cells and enhancing cancer cell metastasis.

**Conclusions:**

In conclusion, the VIM-AS1/miR-655/ZEB1 axis might be a promising target for improving bladder cancer metastasis via an EMT-related mechanism.

## Background

Bladder cancer is of the 9th most common cancers in the world [1–3]. Most bladder cancers (75–80%) are urothelial cell cancers originating from the epithelial lining of the bladder wall [4, 5] and are commonly noninvasive tumors. Noninvasive bladder cancer patients were usually treated by telescopic removal of the cancer and followed by chemotherapy [6]. Aggressive bladder tumors patients were treated with cystectomy, chemotherapy, or radiotherapy [6]. However, aggressive bladder tumors can lead to worse prognosis in patients, with a 5-year survival rate of only 50% [7]. The molecular mechanisms of bladder cancer metastasis are not fully understood, causing limited progress in improving the survival rate.

Epithelial–mesenchymal transition (EMT), during which epithelial cells lose their epithelial properties and acquire mesenchymal properties, such as invasiveness and mobility [8], is associated with bladder cancer metastasis [9, 10]. E-cadherin, which mediates the cell-to-cell junctions between adjacent epithelial cells [11], is a pivotal tumor eliminator that inhibits the aggressiveness of cancer cells [12, 13]. The loss of E-cadherin, accompanied by the expression of the mesenchymal intermediate filament protein Vimentin [14], is involved in bladder tumorigenesis [15, 16]. In addition, the EMT is also regulated by a cluster of zinc-finger transcription factors, including Snail1, ZEB-1, Snail2, and ZEB-2, through direct binding to the promoter of cadherin 1 (*CDH1*), consequently inhibiting the *CDH1* expression level [17]. The expression of Snail and Slug may be facilitated by transforming growth factor-beta (TGF-β) family members, which are effective mediators of the process of EMT in cancer cells [18, 19]. Uncovering the underlying mechanisms of the deregulation of these related factors is indispensable for developing effective therapies for metastatic bladder cancer.

**Fig. 1:**
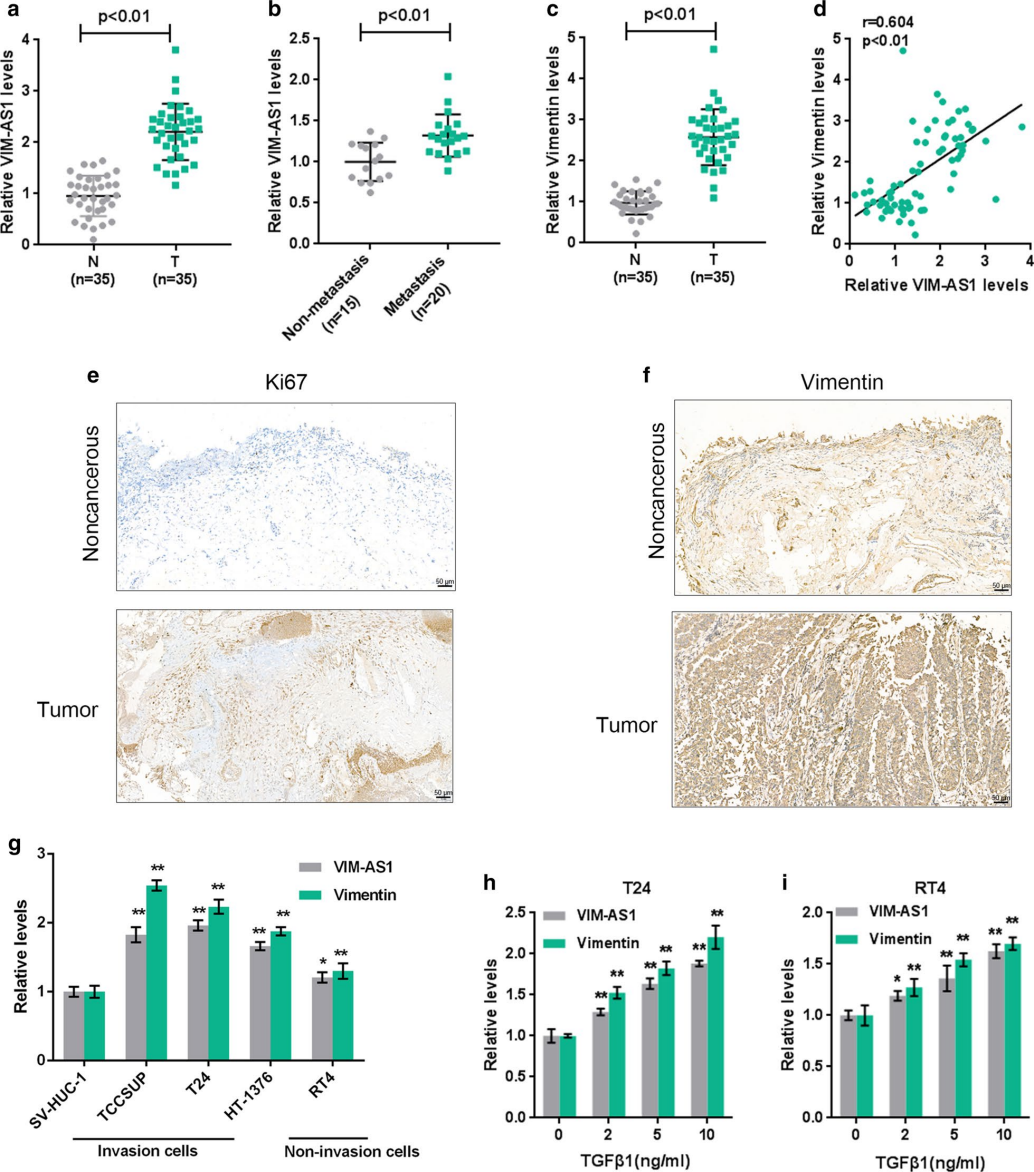
LncRNA VIM-AS1 is upregulated in bladder cancer and is related tobladder cancer metastasis. **a**, **b** The expression of lncRNA VIM-AS1 was determined in 35 paired bladder cancer and noncancerous tissue samples, and analyzed in 15 nonmetastatic and 20 metastatic bladder cancer tissues. **c** Vimentin expression was determined in 35 paired bladder cancer and noncancerous tissue samples by RT-PCR. **d** The correlation of VIM-AS1 and Vimentin expression in tissue samples was analyzed using Pearson’s correlation analysis. **e**, **f** The expression of Ki67 and Vimentin in tissue samples were determined by IHC staining (×200). **g** The expression of VIM-AS1 and Vimentin was determined in a normal cell line, SV-HUC-1, three high-metastatic bladder cancer cell line (TCCSUP, HT-1376, and T24 and a low-metastatic bladder cancer cell line RT4 using real-time PCR. **h**, **i** T24 and RT24 cells were stimulated with 0, 2, 5 and 10 ng/ml TGFβ1 and examined for the expression of VIM-AS1 and Vimentin using real-time PCR. *P < 0.05, **P < 0.01

Long noncoding RNAs (lncRNAs) are a general class of non-protein coding RNAs that are no shorter than 200 nucleotides [20]. Typically, lncRNAs interact with miRNAs [21] to play crucial administrative roles in various organismal processes such as cell growth, development, and tumorigenesis [22–24]. Based on TCGA online data, we investigated lncRNAs that are negatively correlated with *CDH1* expression but positively correlated with *VIM* and Snail expression (Additional file 1: Fig. S1A). Among a total of 14 candidate lncRNAs, higher levels of SLC16A1-AS1, VIM-AS1, or ZEB2-AS1 were associated with a worse prognosis in bladder cancer patients (Additional file 1: Fig. S1F). More importantly, VIM-AS1 expression was the most upregulated in tumor tissues, especially in metastatic tumor tissues (Additional file 1: Fig. S1B–E, Fig. 1a, b). Thus, we speculate that VIM-AS1 may exert a pivotal effect in bladder cancer cell EMT and metastasis. The mutual effects of miRNAs and lncRNAs result in a decrease in the targeted lncRNAs and play significant roles in target gene regulation [21, 25]. Reportedly, miR-655 is an EMT-inhibiting miRNA that targets TGFBR2 and ZEB1 at the same time in cancers [26]. Since VIM-AS1 was predicted by an online tool to target miR-655, herein, we hypothesized that VIM-AS1 might act as a competing endogenous RNA (ceRNA) for miR-655 to offset miR-655-mediated inhibition of ZEB1, therefore modulating bladder cancer cell EMT.

Herein, we monitored the expression levels of VIM-AS1 and Vimentin in high- and low-metastatic bladder cancer cells in response to TGF-β1. By silencing VIM-AS1 in high-metastatic bladder cancer cells and overexpressing VIM-AS1 in low-metastatic bladder cancer cells, we investigated the detailed cellular functions of VIM-AS1 on the migration and invasion ability of bladder cancer cells. Next, the predicted interactions between miR-655 and VIM-AS1 and ZEB1 were validated, and functional effects of VIM-AS1, miR-655 and ZEB1 on bladder cancer cell migration and invasion were evaluated. In summary, we provide a novel lncRNA-miRNA interaction-based mechanism of EMT regulation in bladder cancer cells.

## Materials and methods

### Clinical tissue samples

Human bladder cancer and noncancerous bladder specimens were obtained from Xiangya Hospital with informed consent under the approval of the Xiangya Hospital’s Protection of Human Subjects Committee (No. 201803776). A total of 35 paired bladder urothelial carcinoma and noncancerous clinical tissue (2 cm from the tumor edge) samples were acquired from patients who undergo surgical resection in Xiangya Hospital.

### Cell lines and cell culture

A normal human uroepithelium cell line, SV-HUC-1 (ATCC® CRL-9520™) was obtained from American Type Culture Collection (ATCC, Manassas, VA, USA) and cultured in F-12K medium (Catalog No. 30-2004, ATCC). High-metastatic bladder cancer cell line, TCCSUP (G4; ATCC® HTB-5™), T24 (G3; ATCC® HTB-4™), and HT-1376 (G3; ATCC® CRL-1472™), and a low-metastatic bladder cancer cell line, RT4 (G1; ATCC® HTB-2™) were obtained from ATCC. TCCSUP and HT-1376 cells were cultured in EMEM medium. T24 and RT4 cells were cultured in McCoy’s 5a medium complemented with 10% fetal bovine serum (FBS) (Invitrogen, USA). All cells were incubated at 37 °C in a humidified atmosphere with 5% CO_2_.

### Cell transfection

To knock down or overexpress VIM-AS1, we transfected target cell lines with si1-VIM-AS1/si2-VIM-AS1 or VIM-AS1-overexpressing vector (GenePharma, Shanghai, China). To knock down or overexpress ZEB1, we transfected target cell lines with si-ZEB1 or ZEB1-overexpressing vector (GenePharma). To overexpress or inhibit miR-655, we transfected the corresponding miRNA mimics or inhibitor (GenePharma) into the investigated cell lines with the utilization of Lipofectamine™ 2000 (Invitrogen, Waltham, MA, USA) in accordance with the manufacturer’s protocols. Relevant sequences of were shown in Table 1. The transfection efficiency was explored by real-time PCR. The more method details of cell transfection assay listed in Additional file 1.

**Table 1:** The primer sequence

Name	Forward	Reverse
RT-PCR VIM-AS1	CTCAGACCTGTAGCATCAGCATCAC	TTCATTTCCTCATAAGCGAACACTC
RT-PCR ZEB1	AGCAGTGAAAGAGAAGGGAATGC	GGTCCTCTTCAGGTGCCTCAG
RT-PCR Vimentin	AGTCCACTGAGTACCGGAGAC	CATTTCACGCATCTGGCGTTC
RT-PCR GAPDH	ACAGCCTCAAGATCATCAGC	GGTCATGAGTCCTTCCACGAT
RT-PCR miR-655-3p	RT:GTCGTATCCAGTGCGTGTCGTGGAGTCGGCAATTGCACTGGATACGACAAAGAG F: GCCGCATAATACATGGTTAAC	CAGTGCGTGTCGTGGA
RT-PCR U6	CTCGCTTCGGCAGCACA	AACGCTTCACGAATTTGCGT
NC mimics	UUCUCCGAACGUGUCACGUTT	ACGUGACACGUUCGGAGAATT
NC inhibitor	CAGUACUUUUGUGUAGUACAA	
MiR-655 mimics	AUAAUACAUGGUUAACCUCUUU	AGAGGUUAACCAUGUAUUAUUU
MiR-655 inhibitor	AAAGAGGUUAACCAUGUAUUAU	
Si-VIM-AS1 1#	GGAUGUGUAAGAGAAUUUATT	UAAAUUCUCUUACACAUCCTT
Si-VIM-AS1 2#	CCAUGUGUGCGAUUCACAATT	UUGUGAAUCGCACACAUGGTT
Si-ZEB1	CCUAGUCAGCCACCUUUAATT	UUAAAGGUGGCUGACUAGGTT
Si-NC	UUCUCCGAACGUGUCACGUTT	ACGUGACACGUUCGGAGAATT
Wt-VIM-AS1	aattctaggcgatcgctcgagAGATTTTAAAATAGAGCATGCTTAATATACTT	attttattgcggccagcggccgcCACTAGTACACCCCCGACGTG
Mut-VIM-AS1	AGTAtgcgctGTATTGGCTGTCCCACATTTTGA	GCCAATACagcgcaTACTACTAAGTAATAAATCTATGTCATCCAAAGG
Wt-ZEB1 3′UTR	aattctaggcgatcgctcgagATTTGGCTCATAACTGTTTCCAAA	attttattgcggccagcggccgcTTTCATACTAAAATATATTATTGTATTAATACAAACTAC
Mut-ZEB1 3′UTR	AgcgcgcaTACCCTTCTTACTGACATATGTACTTTTAGTT	AGAAGGGTAtgcgcgcTGCCAGGTTTGAAGACATACAGTAC
Lv-VIM-AS1 overexpression	ctaccggactcagatctcgagTTCTCCCGGAGGCGCATG	gtaccgtcgactgcagaattcTTTTTTTTTAGCATATCCAAAAATGG
Lv-miR-655-3p overexpression	ctaccggactcagatctcgagATATTTTTAATGGAACCTGCCTTAGA	gtaccgtcgactgcagaattcACAGCAAACACAGCAAAGCAAC
ZEB1 overexpression	ctaccggactcagatctcgagATGAAAGTTACAAATTATAATAC	gtaccgtcgactgcagaattcTTAGGCTTCATTTGTCTTTTC

### Real‐time PCR analyses

RNA extraction was conducted by employing TRIzol™ (Invitrogen), following the methods described before [27] with SYBR Green PCR Master Mix (Qiagen) for mRNA expression level determination and a Hairpin-it miRNAs qPCR kit (GenePharma) for miRNA expression level determination. The expression levels of GAPDH and RNU6B were used as endogenous controls. The primer sequences for PCR were shown in Table 1. Finally, the data were processed using the 2^−ΔΔCt^ relative expression method. The more method details of real time PCR assay listed in Additional file 1.

### Immunofluorescence (IF) staining

IF staining was applied to detect the Vimentin expression as described in the previous method [28]. Cells were cultured in 35 mm glass-bottom dishes. After transfection and/or stimulation, cells were fixed with 4% paraformaldehyde for 15 min, permeabilized with 0.5% Triton X-100 for 10 min, and blocked with 10% normal goat serum for 30 min. Then cells were incubated with a primary antibody against Vimentin (1:250, ab92547, Abcam, Cambridge, MA, USA) at 4 °C overnight. After rinsing with PBS in three times for 5 min each, the cells were incubated with the fluorescent-labeled secondary antibody in the dark for 1 h. Finally, the nuclei were counterstained with DAPI followed by examination and imaging using a fluorescence microscope.

### Protein levels determined by immunoblotting

The protein expression of ZEB1, Vimentin, E-cadherin, and Snail were investigated by using Immunoblotting assays following the methods described before [27]. Briefly, cells were lysed by RIPA buffer. The protein concentration was determined by bicinchoninic acid (BCA) protein kit (Beyotime, China). Next, 30–50 µg proteins were separated by 10% SDS-PAGE minigel and transferred onto an NC membrane. After blocking by 5% non-fat milk in TBST, the membranes were incubated with the primary antibodies: anti-ZEB1 (1:500, ab203829, Abcam), anti-Vimentin (1:1000, ab92547, Abcam), anti-Snail (1:1000, ab53519, Abcam), anti-E-cadherin (1:50, ab1416, Abcam), and anti-GAPDH (1:500, ab8245, Abcam) and an HRP-conjugated secondary antibody. Protein expression signals were visualized through enhanced chemiluminescence (ECL) substrate (Millipore, MA, USA) using GAPDH as an endogenic control. ImageJ software (NIH) was used to calculate the band gray intensity.

### Immunohistochemistry (IHC) staining

Ki67 and Vimentin protein expression in tissue samples were determined by IHC as described in the previous method [29]. The carcinoma and non-cancerous tissue sections were randomly selected and fixed in acetone for 10 min at − 20 °C, permeabilized with 0.2% Triton (Sigma) for 10 min at room temperature, incubated with a blocking solution (3.75% BSA/5% goat serum, Zymed, Carlsbad, CA, USA) for 30 min, and incubated for 2 h with anti-Vimentin (1:200, ab92547, Abcam), anti-Ki67 (1:150, ab1667, Abcam) and anti-ZEB1 (1:100, ab203829, Abcam). All sections were incubated with goat-anti-rabbit HRP-labeled secondary antibodies, then incubated in DAB reagent (Beyotime, China), and subsequently stained with hematoxylin (Beyotime, China). The sections were observed under microscopy (Olympus, Tokyo, Japan).

### Transwell assay

Transwell compartments with 8 µm pores (Millipore, Billerica, MA, USA) were used to detect the invasive capacity of bladder cancer cells c in vitro as described in the previous method [30]. A total of 4 × 10^4^ bladder cancer cells in serum-free medium were placed in the upper chamber on the coated membrane which coated with growth factor reduced Matrigel, and the lower chamber was filled with 10% FBS to prompt bladder cancer cells to pass through the membrane. After incubation for 24 h, cells on the upper surface of the membrane were removed by wiping with a Q-tip, and the invaded cells were fixed with formaldehyde and stained with 0.1% crystal violet. The numbers of invaded cells were calculated in five randomly selected fields under a microscope.

### Wound healing assay

The wound healing assay was used to measure the migratory capacity of bladder cancer cells as described in the previous method [30]. Cells were cultured in 6-well plates for 24 h. Next, an artificial wound was created with a sterile 10 µl pipette tip on the confluent cell monolayer. Images were captured at 0 and 24 h following wound generation by utilizing an inverse microscope (Olympus, Tokyo, Japan).

### Luciferase reporter assay

The wild-type or mutant sequence of human ZEB1 3′UTR harboring the predicted miR-655-3p binding sites, or the wild-type or mutant sequence of VIM-AS1 harboring the predicted miR-655-3p binding sites was cloned into a Renilla psiCHECK^TM^-2 plasmid vector (Promega, Madison, WI, USA). These vectors and miRNA mimics or miRNA inhibitor were co-transfected into 293T cells by the aid of Lipofectamine 2000 (Invitrogen, Waltham, MA, USA), respectively. Luciferase liveness was quantified by utilizing the Luciferase Assay System (Promega, Madison, WI, USA) following standard procedures.

### Data statistics and analysis

Experimental data analyses were performed with SPSS20.0 software and are presented as the mean ± standard deviation (SD) of results from experiments repeated independently three or more times. Two-tailed Student’s t-test was performed to calculate the differences between the two groups. One-way ANOVA followed Turkey’s test was applied to analyze the difference between three or more groups. *P*-values < 0.05 were accepted as statistically significant.

## Results

### LncRNA VIM-AS1 is highly‐expressed in bladder cancer and is related to bladder cancer metastasis

To confirm the involvement of lncRNA VIM-AS1 in bladder cancer metastasis, we first determined the expression level of lncRNA VIM-AS1 in 35 paired bladder cancer and noncancerous tissue samples, and analyzed its expression in 15 nonmetastatic and 20 metastatic bladder carcinoma tissues. As presented in Fig. 1a, b, VIM-AS1 expression was upregulated in tumor tissues, especially in metastatic cancer tissue samples. In addition, the expression of Vimentin, a typical mesenchymal marker related to bladder cancer grade and stage [31, 32], was measured in 35 paired bladder cancer and noncancerous normal tissue samples. Consistent with previous studies, Vimentin expression was significantly upregulated in tumor tissues (Fig. 1c), and was positively correlated with VIM-AS1 expression (Fig. 1d). Moreover, the IHC staining showed that the expression of proliferation marker Ki67 and EMT marker Viemntin were both higher in bladder cancer tissue samples compared to noncancerous tissue samples (Fig. 1e, f). In vitro, the expression levels of VIM-AS1 and Vimentin were detected in a normal cell line, SV-HUC-1, high-metastatic bladder carcinoma cell lines (TCCSUP, T24, and HT-1376), and low-metastatic bladder cancer cell line RT4. Consistent with in vivo observations, VIM-AS1 and Vimentin expression levels were dramatically upregulated in carcinoma cells and were more upregulated in high-metastatic cancer cell lines (Fig. 1g). Next, T24 and RT24 cells were induced to undergo EMT by 0, 2, 5, 10 ng/ml TGFβ1 and were examined for the expression of VIM-AS1 and Vimentin. In both cell lines, VIM-AS1 and Vimentin expression levels were prominently occasioned by TGFβ1 incentive in a concentration-dependent manner (Fig. 1h, i), indicating that VIM-AS1 is related to EMT in bladder cancer and cancer metastasis.

### LncRNA VIM-AS1 promotes bladder cancer cell invasion and migration

**Fig. 2:**
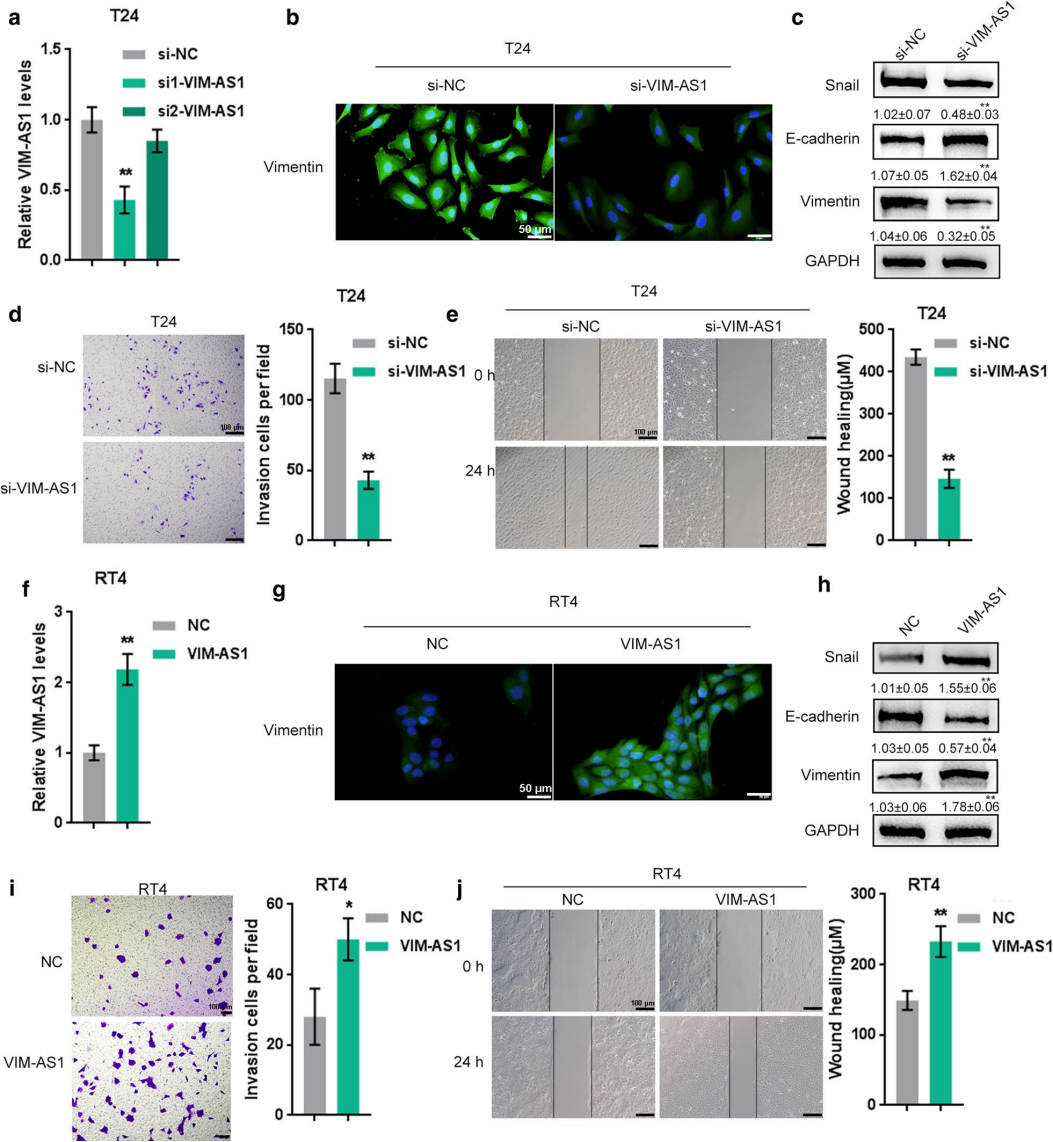
LncRNA VIM-AS1 promotes bladder cancer cell invasion and migration. **a**, **f** VIM-AS1 was knocked down in the high-metastatic bladder cancer cell line T24 by transfection of si1-VIM-AS1 or si2-VIM-AS1 and VIM-AS1 was overexpressed in the low-metastatic bladder cancer cell line RT24 by transfection of the VIM-AS1-overexpressing vector. si-NC or NC vector was used as a negative control. Transfection efficiency was confirmed by real-time PCR. si1-VIM-AS1 was selected for further experiments due to its better transfection efficiency. **b**, **g** The protein content and distribution of Vimentin in T24 and RT24 cells were detected using immunofluorescence (IF) staining (×400). **c**, **h** The protein levels of Snail, E-cadherin and Vimentin in T24 and RT24 cells were determined by immunoblotting. **d**, **i** The invasive ability of T24 and RT24 cells was determined by Transwell assay. Scale bar = 100 µm. **e**, **j** The migratory ability of T24 and RT24 cells was determined by wound healing assay. Scale bar = 100 µm. *P < 0.05, **P < 0.01

To explore the detailed cellular impacts of VIM-AS1 on bladder carcinoma metastasis, we knock down VIM-AS1 in the high-metastatic bladder cancer cell line T24 by transfection of si1-VIM-AS1 or si2-VIM-AS1 (Fig. 2a), and overexpressed VIM-AS1 in the low-metastatic bladder carcinoma cell RT24 by transfection of the VIM-AS1-overexpressing vector (Fig. 2f), as confirmed by real-time PCR. si1-VIM-AS1 was selected for further experiments due to its better transfection efficiency. Next, the effects of VIM-AS1 overexpression and knockdown on EMT markers, including Vimentin, Snail, and E-cadherin were examined in high-metastatic T24 cells and low-metastatic RT24 cells. As revealed by IF staining, VIM-AS1 knockdown in T24 cells attenuated the fluorescence intensity if Vimentin protein (Fig. 2b), while VIM-AS1 overexpression in RT24 cells enhanced the fluorescence intensity of Vimentin protein (Fig. 2g). Accordingly, VIM-AS1 knockdown in T24 cells significantly reduced Snail protein and increased E-cadherin and Vimentin protein (Fig. 2c), while VIM-AS1 overexpression in RT24 cells increased Snail protein and reduced E-cadherin and Vimentin protein (Fig. 2h).

After confirming the VIM-AS1-induced changes in EMT marker proteins, we subsequently explored the influence of VIM-AS1 on bladder cancer cell invasion and migration. As shown in Fig. 2d, e, i, j, VIM-AS1 knockdown in T24 cells significantly suppressed the migration and invasion of high-metastatic bladder carcinoma cells, while VIM-AS1 overexpression in RT24 cells promoted the migration and invasion of low-metastatic bladder carcinoma cells.

### LncRNA VIM-AS1 competes with ZEB1 for miR-655 binding to modulate bladder cancer metastasis

**Fig. 3:**
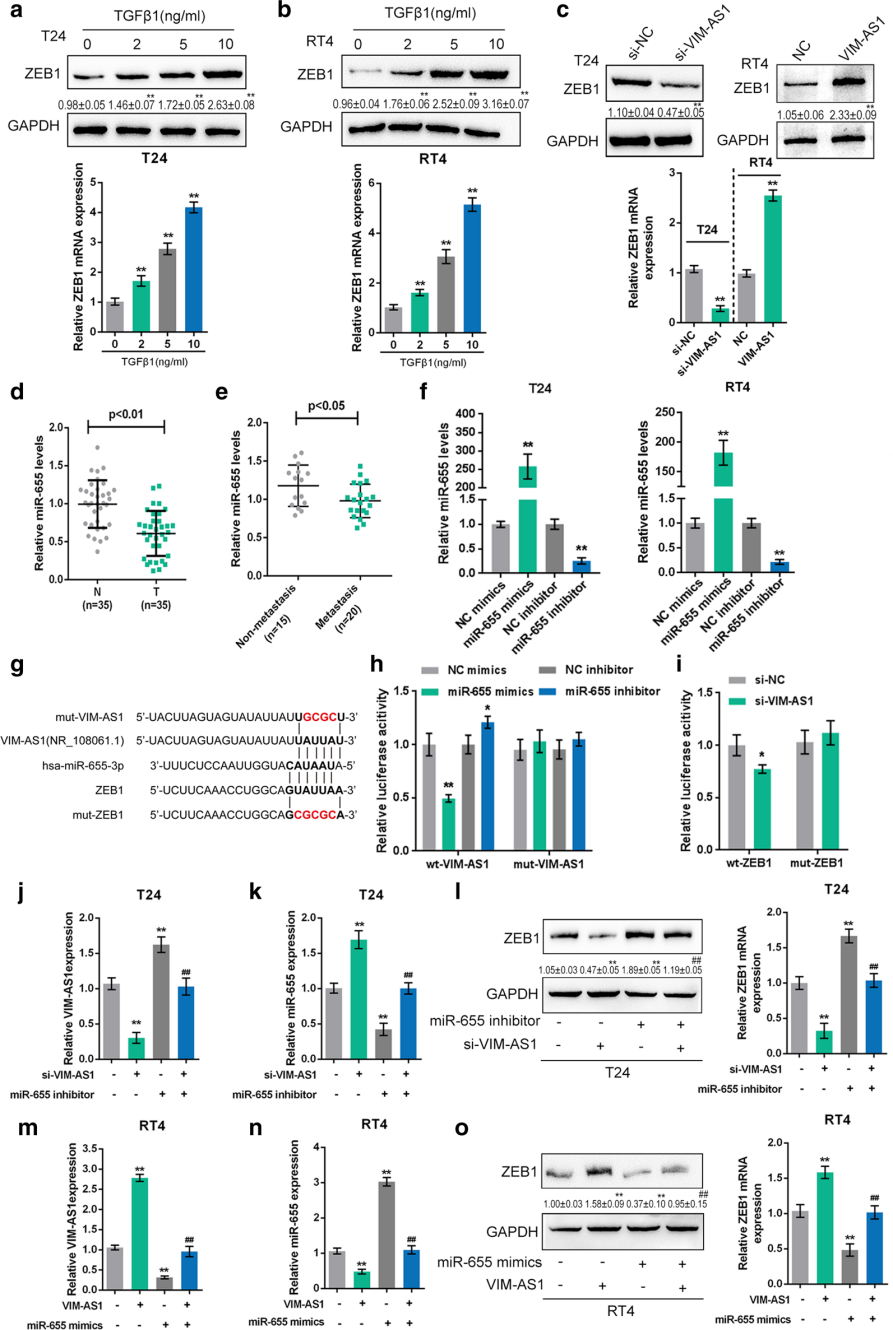
LncRNA VIM-AS1 competes with ZEB1 for miR-655 binding to modulate bladder cancer metastasis. **a**, **b** T24 and RT24 cells were stimulated with 0, 2, 5 and 10 ng/ml TGFβ1 and examined for the protein (upper) and mRNA (under) expression levels of ZEB1. **c** T24 cells were transfected with si-VIM-AS1 and RT24 cells were transfected with VIM-AS1 vector and examined for the protein (upper) and mRNA (under) levels of ZEB1. **d** The expression of miR-655 was determined in 35 paired bladder cancer and noncancerous tissue samples. **e** The expression of miR-655 was analyzed in 15 nonmetastatic and 20 metastatic bladder cancer tissues. **f** miR-655 was overexpressed or inhibited in T24 and RT24 cells by transfection of miR-655 mimics or miR-655 inhibitor, as confirmed by real-time PCR. **g** Wild-type and mutant ZEB1 3′UTR and VIM-AS1 luciferase reporter vectors were constructed as described. **h** The wt-VIM-AS1 or mut-VIM-AS1 vector were co-transfected into 293T cells with miR-655 mimics or miR-655 inhibitor and the luciferase activity was determined. **i** The wt-ZEB1 3′UTR or mut-ZEB1 3′UTR vector was co-transfected into 293T cells with si-VIM-AS1, and the luciferase activity was determined. **j**–**l** T24 cells were co-transfected with miR-655 inhibitor and si-VIM-AS1 and examined for the level of VIM-AS1 (**j**), miR-655 (**k**) and expression of ZEB1 protein and mRNA (**l**). **m**–**o** T24 cells were co-transfected with miR-655 mimics and VIM-AS1 vector and examined for the level of VIM-AS1 (**m**), miR-655 (**n**) and expression of ZEB1 protein and mRNA (O). *P < 0.05, **P < 0.01 compared to control, si-NC, NC, NC mimics, NC inhibitor, inhibitor NC + si-NC or mimics NC + vector group. ^##^*P* < 0.01 compared to miR-655 inhibitor + si-NC or miR-655 mimics + vector group

LncRNAs act as ceRNAs whereby miRNAs and lncRNAs regulate each other’s expression through a targeted binding relationship, therefore counteracting miRNA-mediated suppression of downstream mRNAs [21]. Since ZEB1 is one of the zinc-finger transcription factors that directly binds to the promoter of *CDH1*, concomitantly suppressing *CDH1* expression [17], next we investigated whether ZEB1 is related to VIM-AS1 in a miRNA-dependent manner. T24 and RT24 cells were activated by 0, 2, 5 and 10 ng/ml TGFβ1 and examined for the mRNA and protein expression level of ZEB1. In both cell lines, ZEB1 protein (upper) and mRNA (under) levels were significantly induced by TGFβ1 stimulation in a concentration-dependent manner (Fig. 3a, b). In VIM-AS1-silenced T24 cells, ZEB1 protein (upper) and mRNA (under) levels were reduced (Fig. 3c), while in VIM-AS1-overexpressing RT24 cells, ZEB1 protein (upper) and mRNA (under) levels were increased (Fig. 3c), confirming that ZEB1 could be regulated by VIM-AS1 upon TGFβ1 stimulation.

As we have mentioned, miR-655 is an EMT-inhibiting miRNA targeting ZEB1 and TGFBR2 at the same time in cancers [26]. Since VIM-AS1 was predicted by an online tool to target miR-655, we next investigated whether VIM-AS1 competes with ZEB1 for miR-655 binding. Firstly, we had applied real-time PCR assay to determine the expression level of miR-655 in 35 paired bladder cancer and noncancerous tissue samples (Fig. 3d). MiR-655 expression was dramatically down-regulated in tumor tissues when compare to noncancerous tissues. Then, as indicated in Fig. 3e, miR-655 expression was observably down-regulated in metastatic cancer tissue samples. Next, real-time PCR was performed to assess the transfection efficiency of miR-655 mimics or miR-655 inhibitor in T24 and RT24 cells (Fig. 3f). Two types of ZEB1 3′UTR and VIM-AS1 luciferase reporter vectors were constructed as described (Fig. 3g). In 293T cells, the luciferase activity of wt-VIM-AS1 vector was markedly decreased by miR-655 mimics and enhanced by miR-655 inhibitor, while mutation of the predicted miR-655 binding site reversed the changes in luciferase activity (Fig. 3h), suggesting that VIM-AS1 directly targets miR-655 to inhibit its expression. Moreover, the wt-ZEB1 vector or mut-ZEB1 vector was co-transfected into 293T cells along with si-VIM-AS1. The results showed that the luciferase activity of the wt-ZEB1 3′UTR vector were markedly inhibited by VIM-AS1 silencing, while mutation of the predicted miR-655 binding site abolished the suppression of luciferase activity was abolished (Fig. 3i), suggesting that VIM-AS1 silencing leads to the upregulation of miR-655, therefore suppressing the luciferase activity of the wt-ZEB1 3′UTR vector. As a further confirmation, T24 cells were treated with both the miR-655 inhibitor and si-VIM-AS1, and RT24 cells were treated with both miR-655 mimics and the VIM-AS1 vector. Then, the VIM-AS1 and miR-655 mRNA and ZEB1 mRNA and protein expression levels were quantified. As presented in Fig. 3j, m, VIM-AS1 expression level was notably inhibited by silencing of VIM-AS1 in T24 cells and by miR-655 overexpression in RT24 cells, while markedly promoted by miR-655 inhibition in T24 cells and by overexpression of VIM-AS1 in RT24 cells. As displayed in Fig. 3k, n, miR-655 expression level was dramatically facilitated by knockdown of VIM-AS1 in T24 cells and by overexpression of miR-655 in RT24 cells, but memorably restrained by miR-655 inhibition in T24 cells and by VIM-AS1 overexpression in RT24 cells. As shown in Fig. 3l, o, ZEB1 mRNA (right) and protein (left) expression could be reduced by VIM-AS1 silencing in T24 cells and by miR-655 overexpression in RT24 cells, but increased by miR-655 inhibition in T24 cells and by VIM-AS1 overexpression in RT24 cells. Moreover, the effects of VIM-AS1 silencing or VIM-AS1 overexpression could be significantly reversed by miR-655 inhibition or miR-655 overexpression (Fig. 3l, o), indicating that VIM-AS1 competes with ZEB1 for miR-655 binding to counteract miR-655-mediated ZEB1 suppression.

### Dynamic effects of VIM-AS1 and miR-655 on bladder cancer metastasis

**Fig. 4:**
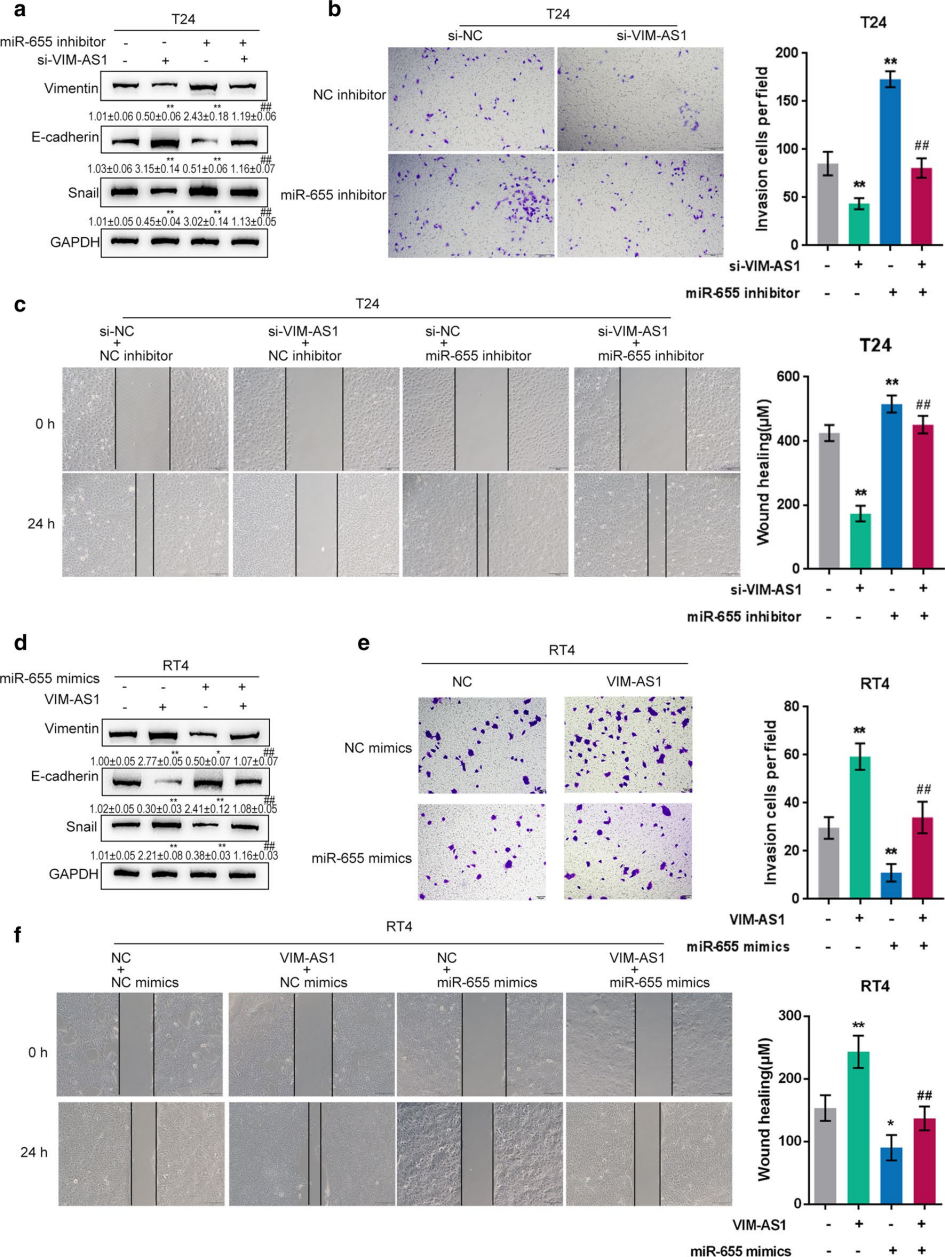
Dynamic effects of VIM-AS1 and miR-655 on bladder cancer metastasis. **a**, **d** T24 cells were co-transfected with si-VIM-AS1 and miR-655 inhibitor; RT24 cells were co-transfected with VIM-AS1 and miR-655 mimics; the protein levels of Vimentin, E-cadherin, and Snail were determined by immunoblotting. **b**, **e** The invasive ability of T24 and RT24 cells was determined by Transwell assay. Scale bar = 100 µm. **c**, **f** The migratory ability of T24 and RT24 cells was determined by wound healing assay. Scale bar = 100 µm. *P < 0.05, **P < 0.01 compared to inhibitor NC + si-NC or mimics NC + vector group. ^##^*P* < 0.01 compared to miR-655 inhibitor + si-NC or miR-655 mimics + vector group

To further investigate the interactions mentioned above, we cotransfected T24 cells with si-VIM-AS1 and the miR-655 inhibitor, and cotransfected RT24 cells with VIM-AS1 and miR-655 mimics. In T24 cells, the protein levels of Vimentin and Snail were significantly reduced by VIM-AS1 silence and increased by miR-655 inhibition; in contrast, E-cadherin protein expression was increased by VIM-AS1 silencing and reduced by miR-655 inhibition (Fig. 4a). The effects of VIM-AS1 were significantly reversed by miR-655 inhibition (Fig. 4a). Regarding cellular functions, VIM-AS1 silencing remarkably inhibited, whereas miR-655 inhibition promoted the invasiveness and migration capability of T24 cells; the impacts of VIM-AS1 silencing were significantly reversed by miR-655 (Fig. 4b, c).

In RT24 cells, VIM-AS1 overexpression significantly increased Vimentin and Snail protein but reduced E-cadherin protein, and miR-655 overexpression exerted opposing effects on these three proteins (Fig. 4d). Regarding cellular functions, VIM-AS1 overexpression remarkably promoted, whereas miR-655 overexpression inhibited RT24 cell invasiveness and migration capability; the impacts of VIM-AS1 overexpression on carcinoma cells were significantly abolished by miR-655 overexpression (Fig. 4e, f). These findings implied that VIM-AS1 promoted the migratory and invasive ability of both high-metastatic and low-metastatic bladder cancer cells by targeting miR-655.

### Effects of VIM-AS1 and miR-655 on the growth of xenograft formed in nude mice

**Fig. 5:**
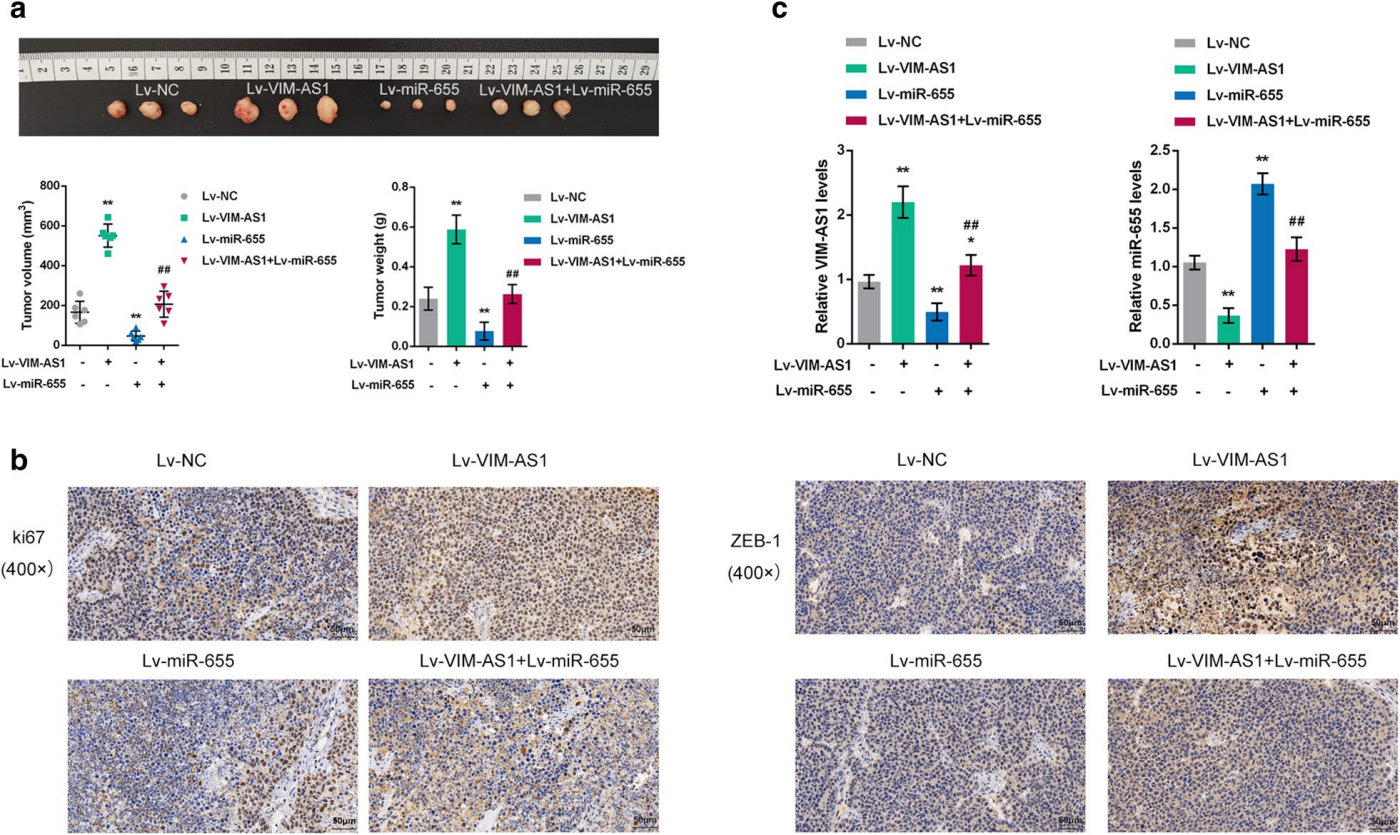
Effects of VIM-AS1 and miR-655 on the growth of xenograft formed in nude mice. The stable T24 cells of Lv-negative control (NC), Lv-VIM-AS1, Lv-miR-655 and Lv-VIM-AS1 + miR-655 were subcutaneous injected into the armpit of nude mice respectively. **a** At the end of the xenograft formed experiment (the 25th day), mice were euthanized and tumor tissues were excised, the tumor volumes, weight and sizes in different experiment groups were detected. **b** IHC staining (×200) was applied to detect the protein expression of proliferation marker Ki67 and ZEB1 in tumor tissues from nude mice. **c** The VIM-AS1 and miR-655 expression levels in tumor tissues from nude mice were determined by real-time PCR assay. *P < 0.05, **P < 0.01 compared to Lv-NC group; ^##^*P* < 0.01 compared to Lv-VIM-AS1 or Lv-miR-655 group

The effect of overexpression of VIM-AS1 and miR-655 towards the growth and tumorigenesis of bladder cancer in vivo was evaluated on xenograft nude mice model. The stable T24 cells of Lv-negative control, Lv-VIM-AS1, Lv-miR-655 and Lv-VIM-AS1 + miR-655 were subcutaneous injected into the armpit of nude mice respectively. At the end of the experiment (the 25th day), mice were euthanized and tumor tissues were excised, the tumor volumes, weight and sizes of xenograft formed by T24 cells with VIM-AS1 overexpression were clearly greater than those formed by normal T24 cells, while the tumor volumes, weight and sizes in Lv-miR-655 group were observably smaller than normal group; VIM-AS1 overexpression could relieves the inhibition effect of miR-655 on the growth and tumorigenesis of bladder cancer (Fig. 5a). Then, the IHC staining was applied to detect the protein expression of proliferation marker Ki67 and ZEB1 in tumor tissues from nude mice (Fig. 5b). The results showed that the overexpression of VIM-AS1 dramatically facilitated Ki67 and ZEB1 levels, while overexpression of miR-655 notably inhibited Ki67 and ZEB1 levels tumor tissues. Moreover, the VIM-AS1 and miR-655 expression levels in tumor tissues from nude mice were also determined by real-time PCR assay (Fig. 5c). The VIM-AS1 expression level was markedly increased in Lv-VIM-AS1, while was memorably decreased in Lv-miR-655 group. By contrast, the miR-655 expression level was significantly decreased in Lv-VIM-AS1, while was prominently increased in Lv-miR-655 group. These data indicate that VIM-AS1 overexpression promoted the growth and tumorigenesis of bladder cancer in vivo, while miR-655 emerged the opposite effect.

### Dynamic effects of VIM-AS1 and ZEB1 on bladder cancer metastasis

**Fig. 6:**
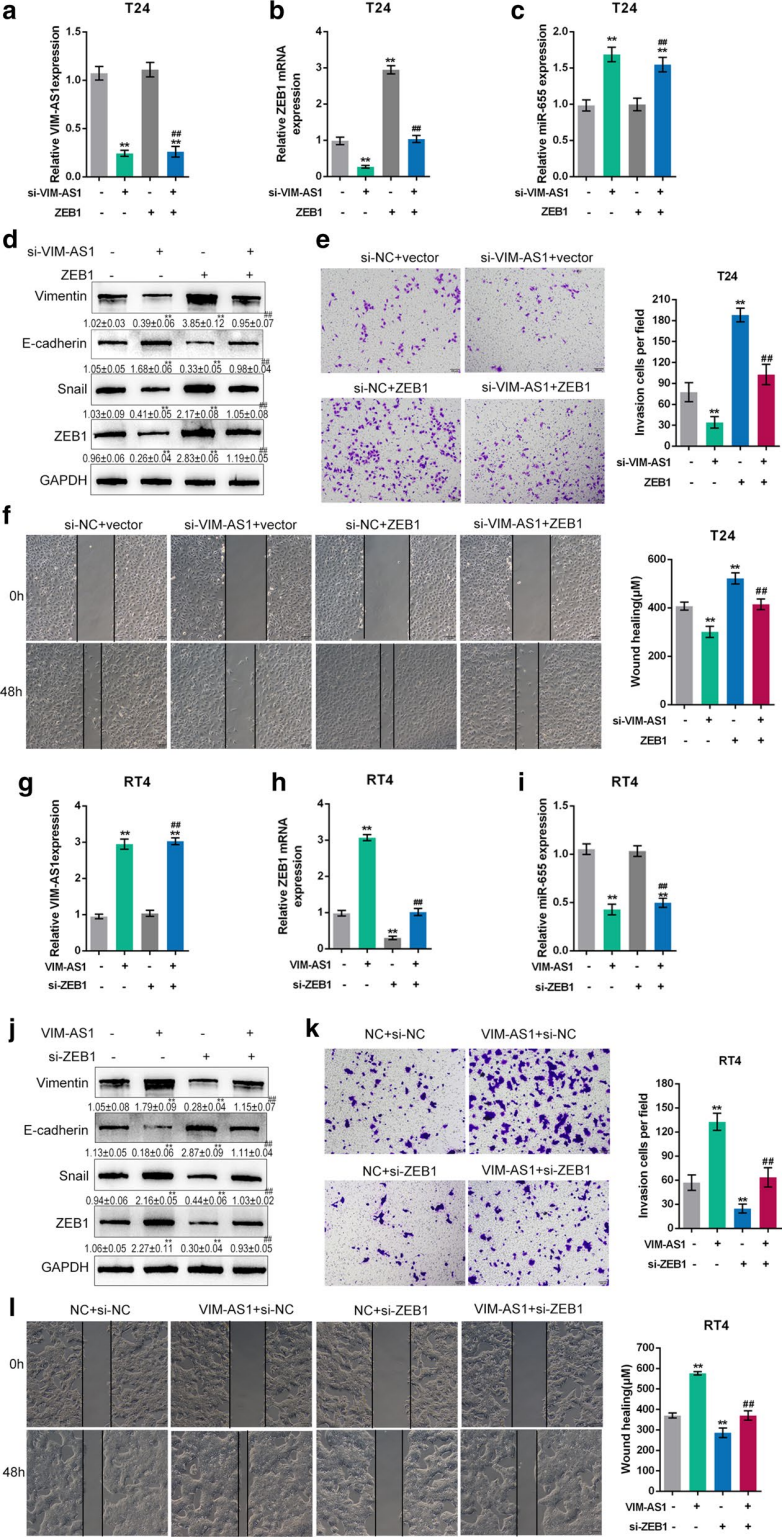
Dynamic effects of VIM-AS1 and ZEB1 on bladder cancer metastasis. **a**–**c** T24 cells were cotransfected with si-VIM-AS1 and ZEB1 vector and the expression levels of VIM-AS1 (**a**), ZEB1 (**b**) and miR-655 (**c**) were detected by real-time PCR analysis. **d**, **j** The protein levels of Vimentin, E-cadherin, Snail and ZEB1 were determined by immunoblotting in T24 and RT24 cells. **e**, **k** The invasive ability of T24 and RT24 cells was determined by Transwell assay. Scale bar = 100 µm. **f**, **l** The migratory ability of T24 and RT24 cells was determined by wound healing assay. Scale bar = 100 µm. **g**–**i** RT24 cells were cotransfected with VIM-AS1 vector and si-ZEB1 and the expression levels of VIM-AS1 (**g**), ZEB1 (**h**) and miR-655 (**i**) were detected by real-time PCR assay. **P < 0.01 compared to si-NC + vector group. ^##^*P* < 0.01 compared to si-NC + ZEB1 or vector + si-ZEB1 group

To further explore the combined effects of VIM-AS1 and ZEB1 on bladder cancer metastasis, we cotransfected T24 cells with si-VIM-AS1 and ZEB1, and cotransfected RT24 cells with VIM-AS1 and si-ZEB1. At firstly, the cell transfection efficiency was measured. In T24 cells, knockdown of VIM-AS1 notably inhibited VIM-AS1 and ZEB1, while increased miR-655 expression; overexpression of ZEB1 observably promoted ZEB1 and had no effect on the expression of VIM-AS1 and miR-655 (Fig. 6a–c). The Vimentin, Snail and ZEB1 protein levels were markedly reduced by silencing of VIM-AS1 and increased by ZEB1 overexpression; on the contrary, E-cadherin protein expression was increased by VIM-AS1 silencing and reduced by ZEB1 overexpression (Fig. 6d). Moreover, silencing of VIM-AS1 prominently inhibited, whereas ZEB1 overexpression promoted the invasiveness and migration capability of T24 cells (Fig. 6e, f). The effects of VIM-AS1 to EMT-related protein expression and invasiveness and migration capability of T24 cells were dramatically eliminated by overexpression of ZEB1.

In RT24 cells, overexpression of VIM-AS1 markedly promoted VIM-AS1 and ZEB1, while restrained miR-655 expression; knockdown of ZEB1 memorably suppressed ZEB1 and had no effect on the expression of VIM-AS1 and miR-655 (Fig. 6g–i). Overexpression of VIM-AS1 observably promoted Vimentin, Snail and ZEB1 protein but inhibited E-cadherin protein, and ZEB1 silencing exerted opposing effects on these three proteins (Fig. 6j). Besides, VIM-AS1 overexpression notably increased, whereas ZEB1 silencing restrained RT24 cell invasiveness and migration capability; the stimulative effects of VIM-AS1 overexpression on RT24 cell were markedly relieved by ZEB1 silencing (Fig. 6k, l). All these results proved that VIM-AS1 facilitated the metastasis ability of both high-metastatic and low-metastatic bladder cancer cells by regulating ZEB1 expression.

## Discussion

In the current research, we revealed that lncRNA VIM-AS1 was notably upregulated in bladder carcinoma, especially in metastatic cancer tissues and cell lines. Via serving as a ceRNA for miR-655, VIM-AS1 competed with ZEB1 for miR-655 binding, therefore eliminating miR-655-mediated suppression of ZEB1, finally affecting EMT in both high- and low-metastatic bladder cancer cells and cancer cell metastasis.

Tumor metastasis is the main cause of death of bladder carcinoma patients, and EMT can promote the migratory and invasive capacity of bladder carcinoma [33, 34]. During the EMT process, epithelial marker factors, such as E-cadherin, are decrease, while mesenchymal markers, such as Vimentin, fibronectin and N-cadherin, are increased; furthermore, related transcription factors, including the Twist, ZEB, and Snail families, are stimulated in related cells [35, 36]. In the present research, in addition to the upregulation of VIM-AS1 in bladder cancer tissues, especially in metastatic cancer tissues and high-metastatic cancer cells, the expression of Vimentin was also upregulated, indicating the involvement of VIM-AS1 in EMT progression and metastasis in bladder cancer cells. In high-metastatic T24 cells, VIM-AS1 silencing significantly reduced the protein levels of Vimentin and Snail while increased E-cadherin; in the meantime, the migratory and invasive abilities of T24 cells were inhibited by VIM-AS1 silencing. In contrast, VIM-AS1 overexpression in low-metastatic RT24 cells enhanced the migratory and invasive abilities of RT24 cells by increasing Vimentin and Snail and decreasing E-cadherin. Similarly, Zhang et al. and Bardaji et al. also found that VIM-AS1 modulates the EMT progression of prostate cancer and colorectal cancer [37, 38].

**Fig. 7:**
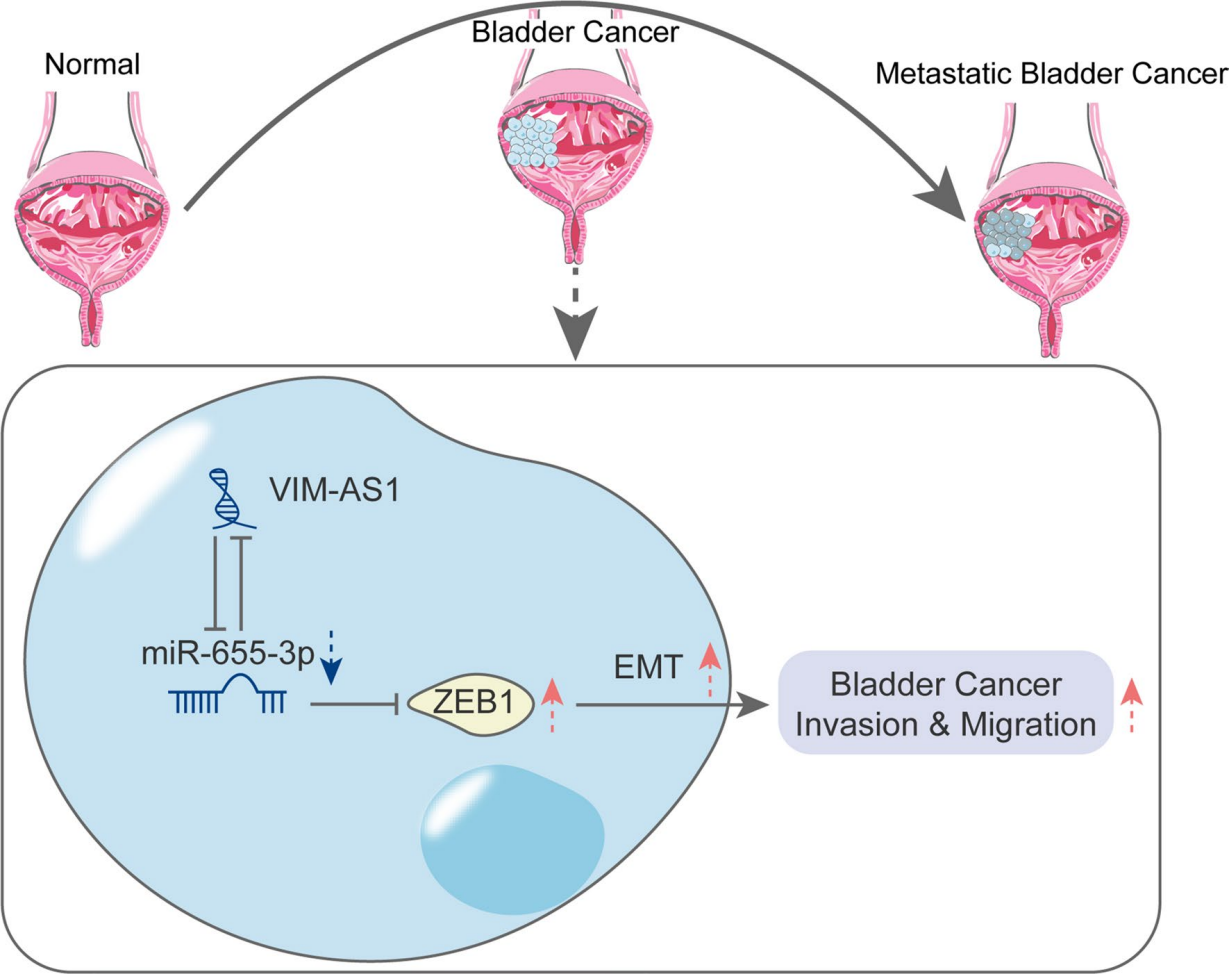
A schematic diagram of the proposed mechanisms of VIM-AS1/miR-655-3P/ZEB1 axis in bladder cancer cells

Alterations of miRNAs expression are implicated in almost all fields of cancer biology, including cell growth, apoptosis, migration and/or invasion [39, 40]. The ceRNA hypothesis indicates that different kinds of RNA molecules (such as lncRNAs, and circRNA) could regulate each other’s expression by emulously suppressing miRNAs via common miRNA response elements (MREs) [41, 42], thereby altering signaling pathways. In bladder cancer, several lncRNA/miRNA axes have acted as regulators of cancer metastasis, including lncRNA H19/miR-29b, lncRNA XIST/miR-124 and lncRNA UCA1/miR-196a [43–45]. In the present study, online tools and experimental analyses indicated that miR-655, an EMT-inhibiting miRNA that targets TGFBR2 and ZEB1 [26], is a downstream target of VIM-AS1. By competing with ZEB1 for miR-655 binding, VIM-AS1 could counteract the miR-655-mediated suppression of ZEB1 in both T24 and RT24 cells. In high-metastatic T24 cells, miR-655 inhibition aggravated cancer cell invasiveness and migration capability by promoting EMT, while miR-655 overexpression in low-metastatic RT24 cells suppressed cancer cell invasiveness and migration capability by inhibiting EMT. More importantly, the effects of VIM-AS1 on both cell lines were significantly reversed by miR-655, indicating that VIM-AS1 acts as a ceRNA to competitively bind to miR-655, therefore rescuing ZEB1 expression and promoting EMT and metastasis in bladder cancer cells (Fig. 7).

## Conclusions

In conclusion, we provide a novel lncRNA-miRNA interaction-based mechanism of EMT regulation in bladder cancer cells. The VIM-AS1/miR-655/ZEB1 axis might be a potential target for improving bladder cancer metastasis via an EMT-related mechanism. However, the in vivo function of VIM-AS1/miR-655/ZEB1 axis in bladder cancer needs further investigation.

## Supplementary Information

**Additional file 1: Figure S1. **Selection of lncRNAs related to bladder cancer metastasis. (A) A schematic diagram showing lncRNAs related to E-cadherin, Vimentin, and Snail expression based on TCGA database. (B–E) The expression of ZEB2-AS1 and SLC16A1-AS1 in normal and bladder cancer tissues (nonmetastatic and metastatic) examined and analyzed by real-time PCR. (F) Kaplan–Meier analysis showing the correlation between VIM-AS1 expression and the survival percentage in bladder cancer patients.

## Data Availability

Please contact the authors for data requests.
