# Supplementary figures and images for: The VIM-AS1/miR-655/ZEB1 axis modulates bladder cancer cell metastasis by regulating epithelial–mesenchymal transition
Source: Cancer Cell Int. 2021 Apr 26;21:233. doi: 10.1186/s12935-021-01841-y (PMC8074428; doi:10.1186/s12935-021-01841-y)

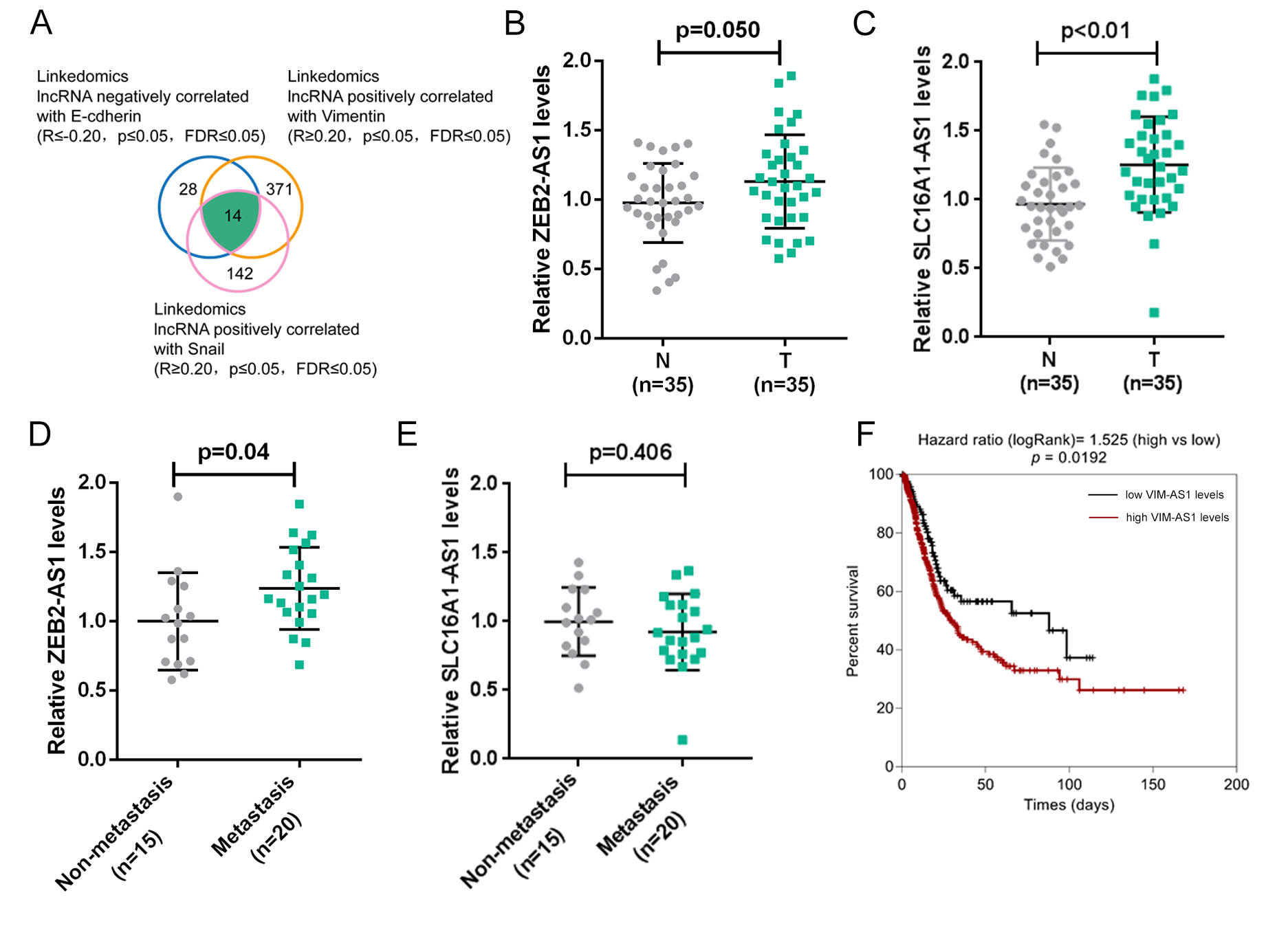

Supplement: Supplementary file 1 — Additional file 1: Figure S1. Selection of lncRNAs related to bladder cancer metastasis. (A) A schematic diagram showing lncRNAs related to E-cadherin, Vimentin, and Snail expression based on TCGA database. (B–E) The expression of ZEB2-AS1 and SLC16A1-AS1 in normal and bladder cancer tissues (nonmetastatic and metastatic) examined and analyzed by real-time PCR. (F) Kaplan–Meier analysis showing the correlation between VIM-AS1 expression and the survival percentage in bladder cancer patients. [file 12935_2021_1841_MOESM1_ESM.tif]
